# Immediate Root Filling

**Published:** 1892-08

**Authors:** S. Hubbell

**Affiliations:** Burlington, Vt.


### Immediate Root Filling.

BY DR. S. HUBBELL, BURLINGTON, VT.

Read before the Vermont State Dental Society, March, 1892.

In preparing a paper on the subject of immediate root filling, it is not my desire to present any new methods of root filling, or in fact to discuss any of the methods now in use. I wish merely to present my reasons for believing that under certain conditions the practice of immediate root filling cannot be successful.

There are many conditions of the roots of teeth presenting themselves for filling, from the healthy root canals through all stages of inflammation, decomposing and dead pulps, complicated with disease of the peridental membrane, and alveolar abscess.

It is due to these conditions that so many men differ in their methods of treatment, in regard to the very important point as to the proper time for permanent filling.

I believe the method of immediate root filling would be universally adopted, if we were only required to fill healthy roots; that is, canals from which healthy or living pulps had been extracted. These are the most favorable cases, as an incised wound is left at the apex of the root which will heal by first intention. The canal may be permanently filled at once with slight danger of irritation following, provided all proper aseptic precautions have been observed.

If the pulp has been destroyed by the use of arsenious acid, and the acid has been confined wholly to the pulp, and its removal is experienced with some pain, we may feel comparatively safe in filling at once.

We have undoubtedly noticed the dentine and enamel of teeth become partially and sometimes thoroughly discolored from the use of copper amalgam with which they have been filled.

If these dense tissues will allow this coloring matter to penetrate them, may we not well believe that septic matter from a pulp having been a source of infection for a long period, and having caused alveolar abscess, will thoroughly infiltrate the dentine and at times reach the peridental membrane. For the tubes, which penetrate the dentine, opening with their largest diameters into the pulp chamber, are, when a tooth is in a normal condition a source of strength and nutrition, but when the tooth is attacked with caries they facilitate its decalcification, and finally decomposition of the organic which has served for a matrix for the inorganic. The tubes in this condition, and the imperfections which are liable to be present in the dentine, invite the free ingress of fluids which consume and disintegrate the organic material of the tooth, and supply as their products a mass of septic and decomposing tooth structure. Under these conditions how can we accept the statement that all roots can be made aseptic by one treatment, the root permanently filled, and without any future trouble, (as it would necessarily be by the immediate root filling method.) The decomposition, which renders the most uncertain and troublesome treatment of any of the pathological conditions of the pulp, requires the ingress of atmospheric germs.

This septic poison, aided by this prime factor, atmospheric air, so inflates the surrounding tissues, that by long standing the most thorough treatment will in some cases prove effectual. It is with this class of diseased roots that we find so wide a difference exists between the immediate root filler and the practitioner with more conservative ideas. Great confidence is placed on nature's curative power by the typical immediate root filler. This power is everywhere present in all parts of the body, yet its power to heal cannot be definitely known.

It is without doubt a safe practice to keep the root canal open until we have more conclusive evidence that a cure has been effected than that of the termination of the discharge. We should look for further results, the evidence of new tissue being formed by granulation at the apex, and the fistula healed. Even at this stage of treatment a positive cure may not be effected. Should any amount of inflammation appear, especially if the patient is in a depleted condition caused by constitutional disturbances, the new tissue formation is liable to break down and we still have a chronic case of abscess. In blind abscess and all other complications which are apt to follow this precipitate method, immediate root filling, the surgical process of opening up the gums and drilling down through the alveolus to the abscess is frequently mentioned as an effective and painless operation. We may treat the abscess through this opening, but not nearly so effectually as through the nerve canal. Besides the uncertainty of affording relief it must be performed at the most painful stage just before the formation of pus.

The papers and discussions which have been presented on this subject in the past, have had, I think, a good effect on our private practice. It has resulted in the adoption of better methods of antisepsis, and we, who have failed in the two extremes of over treatment and immediate filling, will be competent to practice more exact methods, and no doubt in the future have more uniformally good results in our efforts to save pulpless teeth.

